# Supplementary material for: Genomic signatures reveal DNA damage response deficiency in colorectal cancer brain metastases
Source: Nat Commun. 2019 Jul 18;10:3190. doi: 10.1038/s41467-019-10987-3 (PMC6639368; doi:10.1038/s41467-019-10987-3)
Supplement: Supplementary file 1 — Supplementary Information [file 41467_2019_10987_MOESM1_ESM.pdf]

Supplementary information

Genomic Signatures Reveal DNA Damage Response Deficiency in Brain Metastases of Colorectal Cancer

Sun et al.

Supplementary Figure 1. Major mutational signatures in our patients and TCGA CRC patients. TCGA patients were included after matching for age, sex and stage at diagnosis using nearest matching methods. The results were similar with the proportions we provided in Figure 1B.

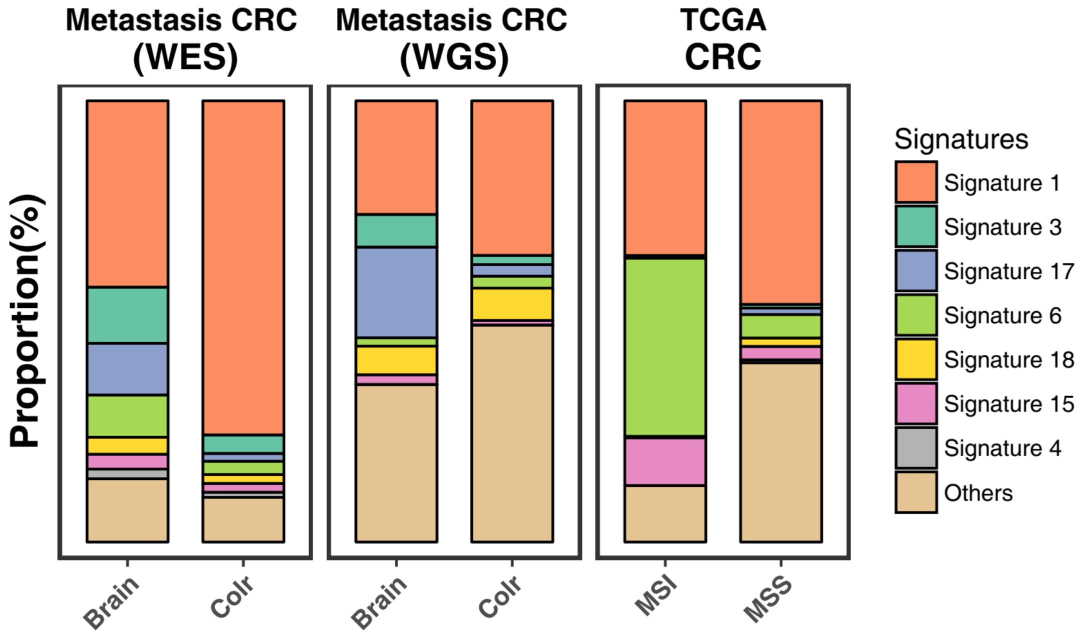

Supplementary Figure 2. The detailed proportion of 4 mutational signatures in all BMs and primary tissues.

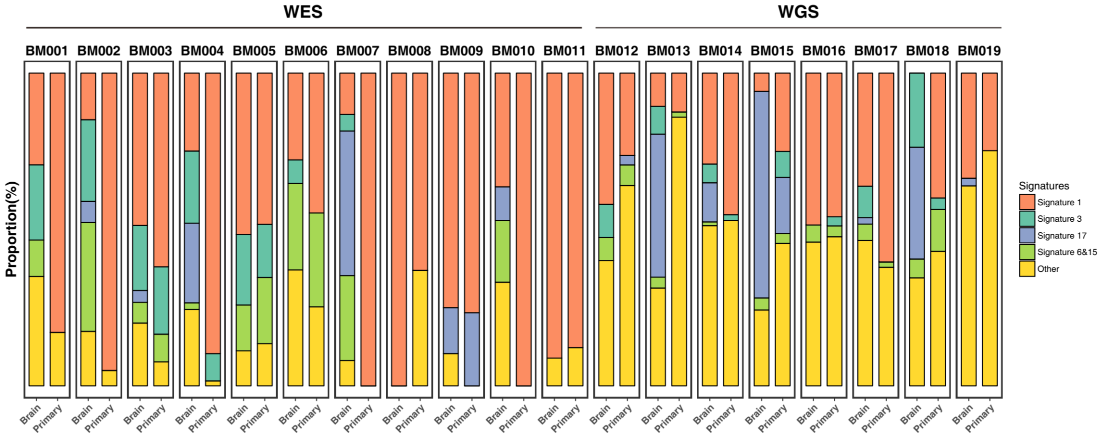

Supplementary Figure 3. GSEA analysis also suggested that MMR and HR gene sets were significantly negatively enriched in brain metastasis tissues. The bottom portion of the figure plots the enrichment scores (ES) for each gene, whereas the top portion of the plot shows the value of the ranking metric moving down the list of ranked genes. (A) Mismatch repair related genes. (B) Homologous recombination related genes.

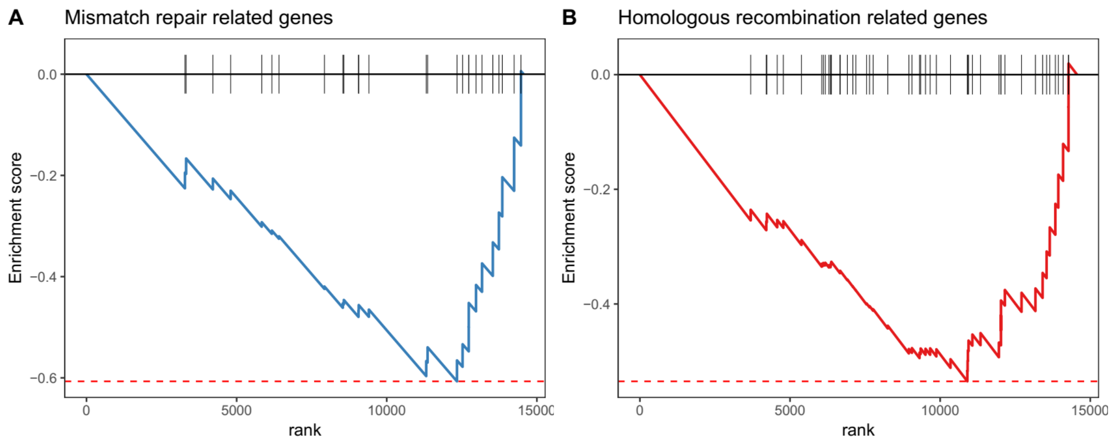

Supplementary Figure 4. Correlation between the proportion of COSMIC Signature 17 and mutation burden. We estimated the proportion of each mutational signature R package deconstructSigs according to the mutation pattern matrix reported by COSMIC. The mutation burden was defined as the number of somatic mutations per megabase (/MB) of genome examined.

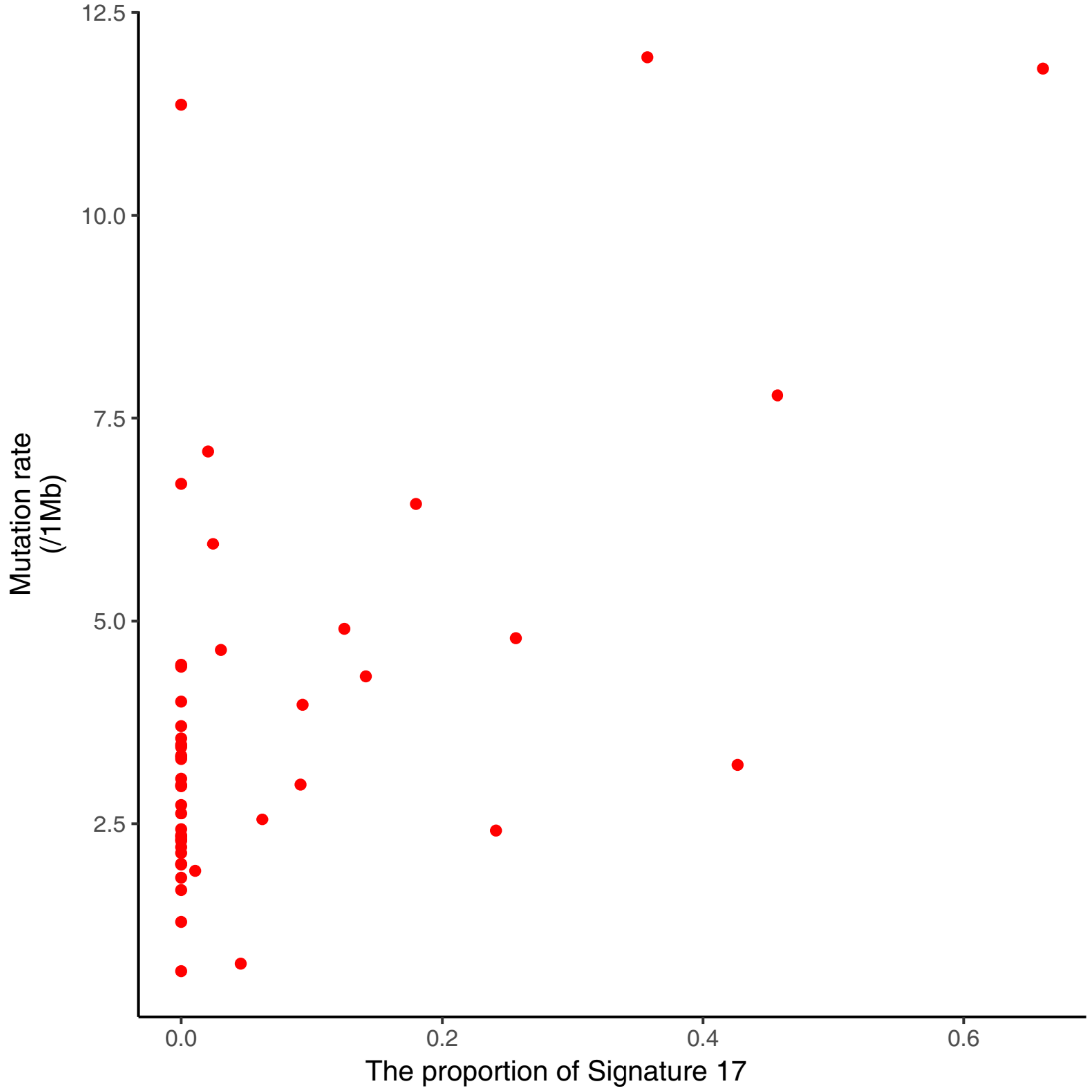

Supplementary Figure 5. No significant difference was found between the COSMIC signature (signature 1, 3, 17 and 6&15) proportion of liver metastasis and primary colorectal tissues. Mutation calling and mutational signature extraction followed the same pipeline in BM "trios".

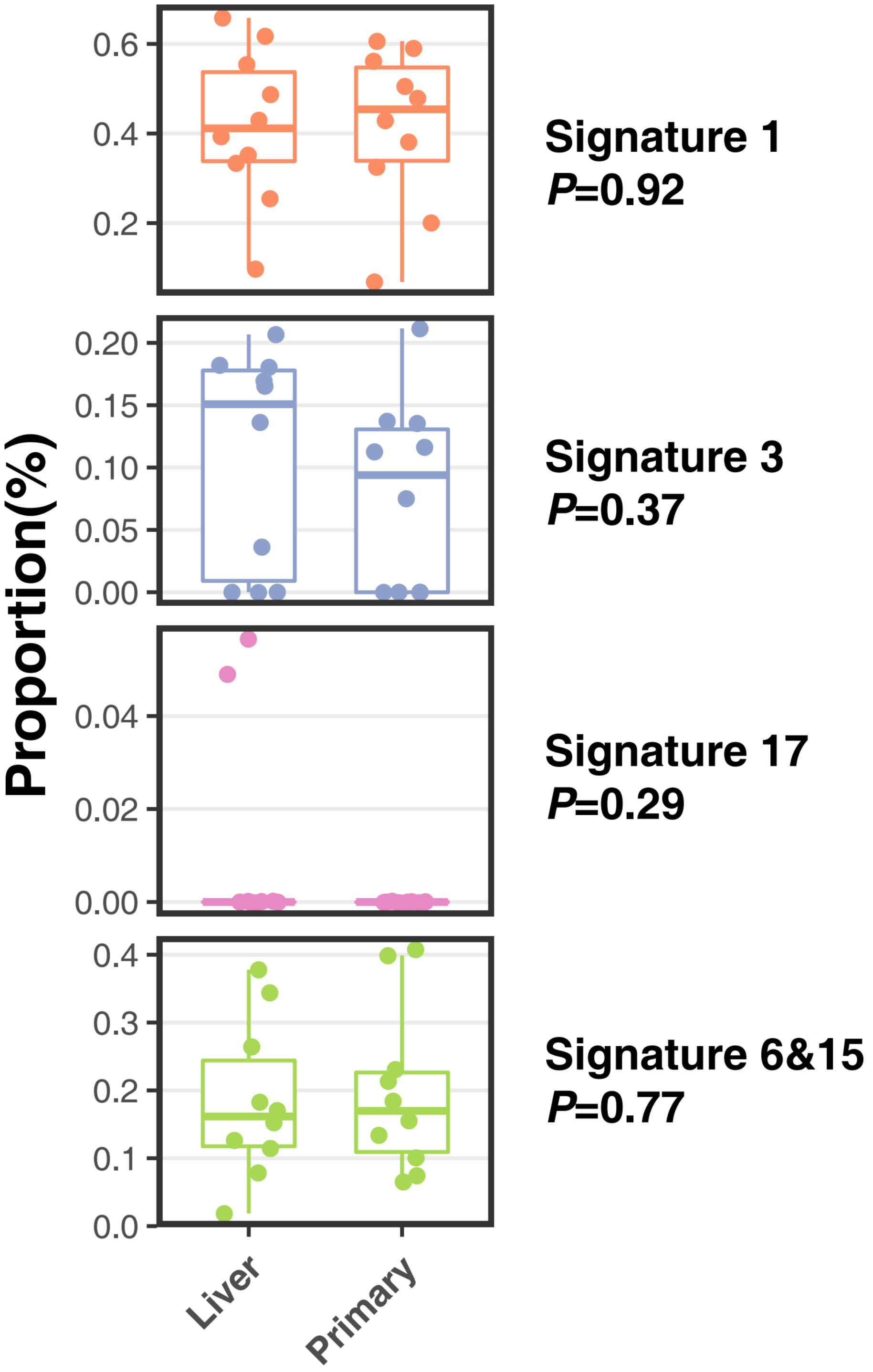

Supplementary Figure 6. Inferred phylogenetic tree of BM and matched primary samples from 7 “trios”. We first estimated the number of subclones of each samples and the relative contribution. Then phylogenetic model was inferred according to subclones estimated. For each sample, subclones are presented in different colors and the samples carried the subclones are presented next to the subclone number. * indicates the founding clones of each samples. The founding mutations of brain metastasis and TP53 mutations are presented next to the initiated subclones.

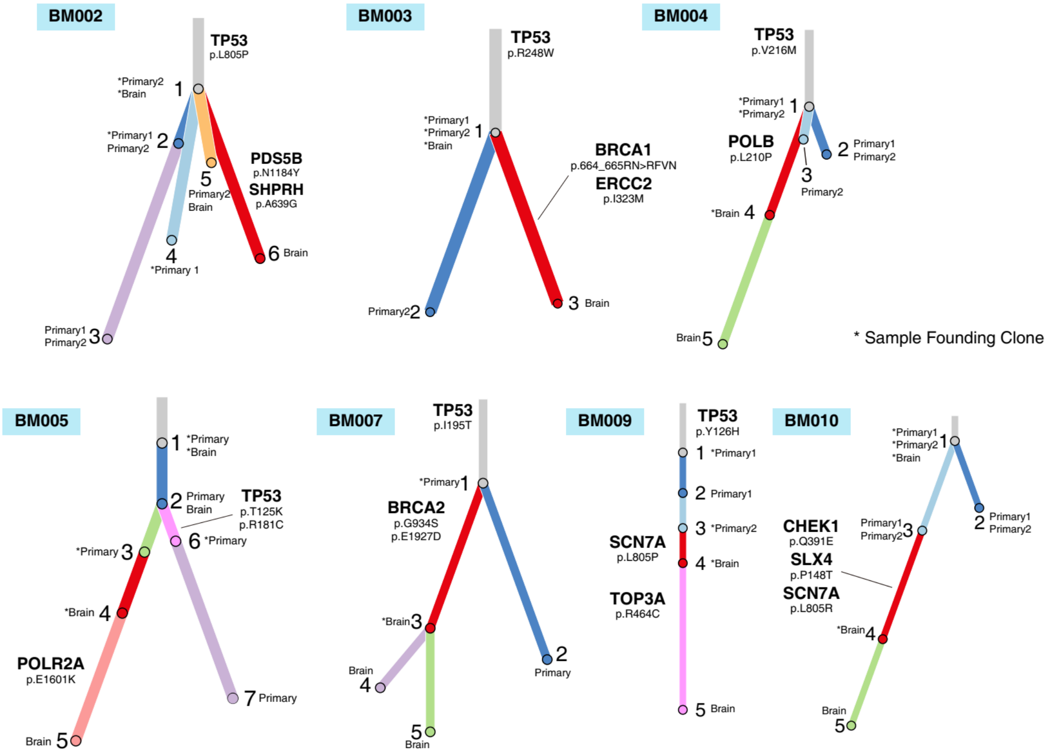

Supplementary Figure 7. Description of DDR mutations.

A. Mutational lolliplot of brain-specific mutation in *BRCA1* for patient BM001. For each gene, the mutations of our study (up) and COSMIC (down) were presented as a circle with different colors according to their function annotation. The protein domains are presented according to Pfam databases (https://pfam.xfam.org/). The fractions of reads with alternative reads are presented to show the biallelic loss of the BRCA1 mutation.

B. Mutational lolliplot of brain-specific mutation in *BRCA2* for patient BM007.

C. Proportion difference of Signature 3 between brain metastases and primary tissues from patients BM001, BM002, BM004, BM007, BM013, BM014, and BM018.

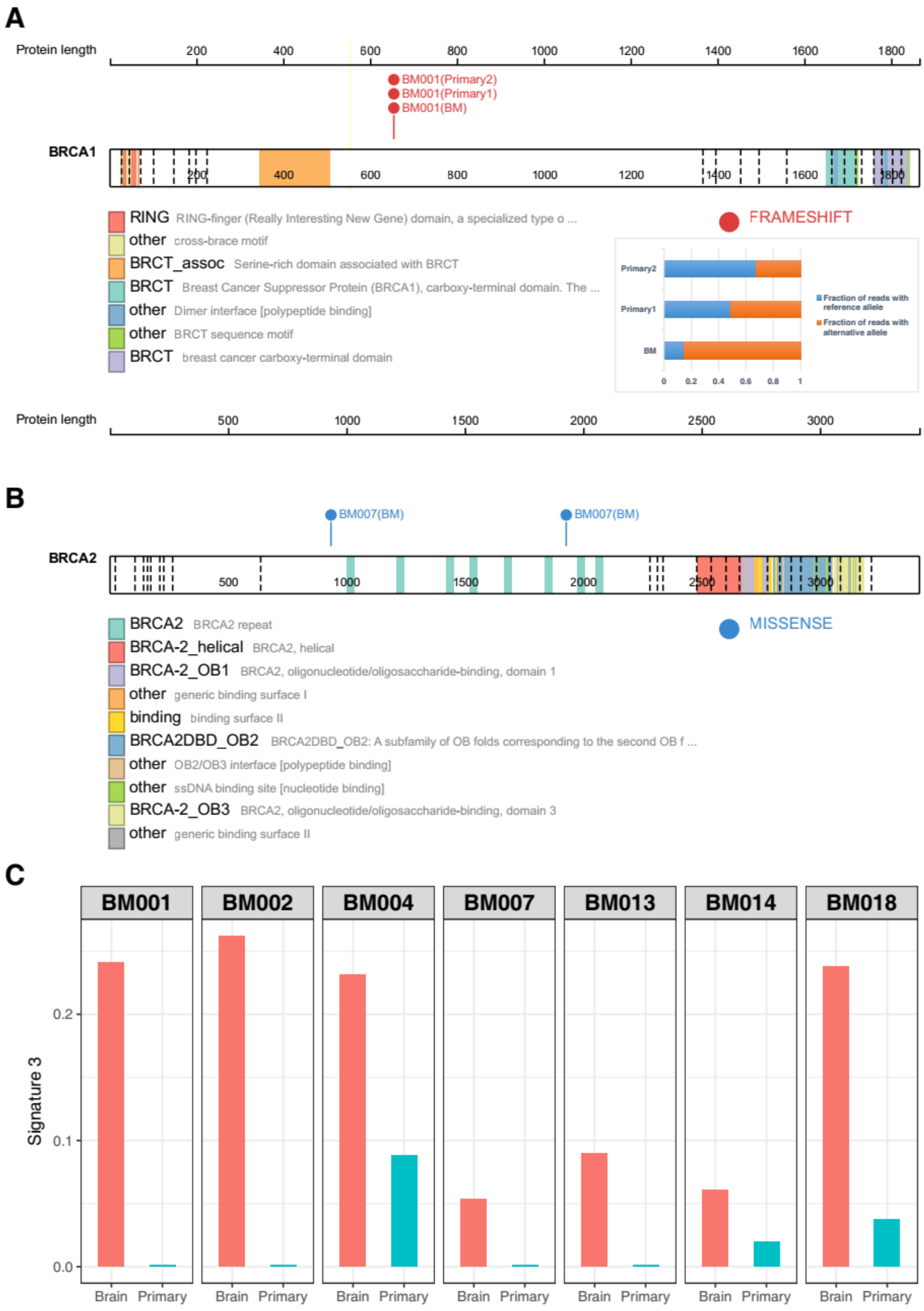
A
Protein length
BM001(Primary2)
BM001(Primary1)
BM001(BM)
BRCA1
RING RING-finger (Really Interesting New Gene) domain, a specialized type o ...
other cross-brace motif
BRCT_assoc Serine-rich domain associated with BRCT
BRCT Breast Cancer Suppressor Protein (BRCA1), carboxy-terminal domain. The ...
other Dimer interface [polypeptide binding]
other BRCT sequence motif
BRCT breast cancer carboxy-terminal domain
FRAMESHIFT
Primary2
Primary1
BM
Fraction of reads with reference allele
Fraction of reads with alternative allele
B
BM007(BM)
BM007(BM)
BRCA2
BRCA2 BRCA2 repeat
BRCA-2_helical BRCA2, helical
BRCA-2_OB1 BRCA2, oligonucleotide/oligosaccharide-binding, domain 1
other generic binding surface I
binding binding surface II
BRCA2DBD_OB2 BRCA2DBD_OB2: A subfamily of OB folds corresponding to the second OB f ...
other OB2/OB3 interface [polypeptide binding]
other ssDNA binding site [nucleotide binding]
BRCA-2_OB3 BRCA2, oligonucleotide/oligosaccharide-binding, domain 3
other generic binding surface II
MISSENSE
C
BM001
BM002
BM004
BM007
BM013
BM014
BM018
Signature 3
Brain
Primary

Supplementary Figure 8. Twenty-seven potential driver genes related to BM. In this analysis, we included TCGA patients after matching for age, sex and stage at diagnosis using nearest matching from MatchIt R package to avoid the influence of the covariates. In total, 152 TCGA patients corrected for age, sex and stage were used for mutation rate calculation. The results were similar to the mutation rates we provided in Figure 4A.

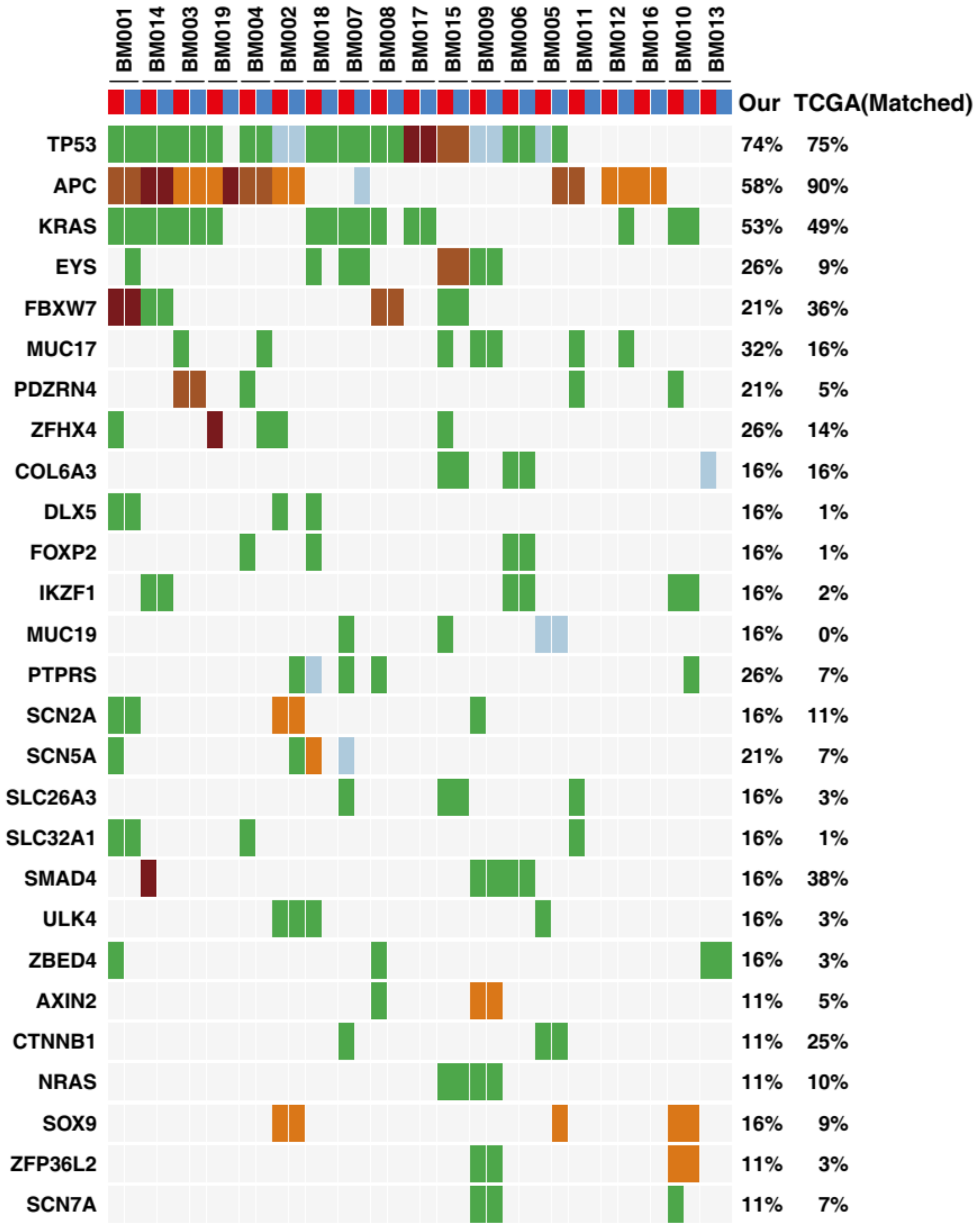

Supplementary Figure 9. A. *IKZF1* is down-regulated in TCGA CRC bulk tissues. B. *IKZF1* is highly expressed in 4.0% of CRC tumor cells and in none of the normal mucosa cells.

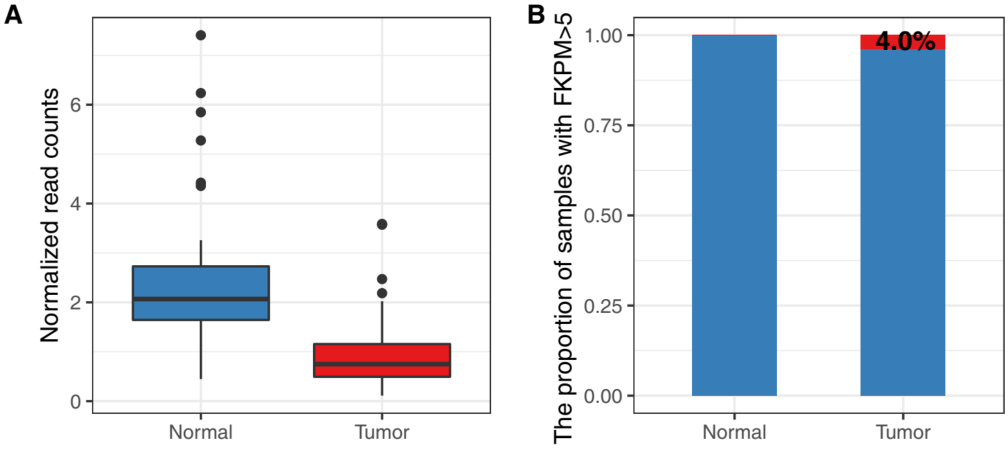

Supplementary Figure 10. Expression of *SLC32A1* in multiple normal tissues from The Genotype-Tissue Expression (GTEx, Version V7).

A

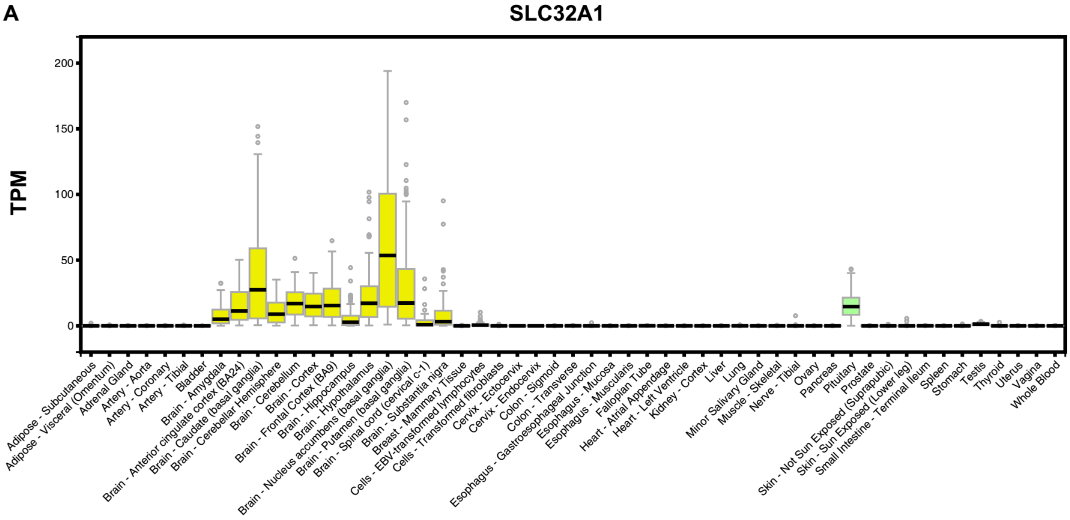

Supplementary Figure 11. Expression of *PDZRN4* in multiple normal tissues from GTEx (Version V7).

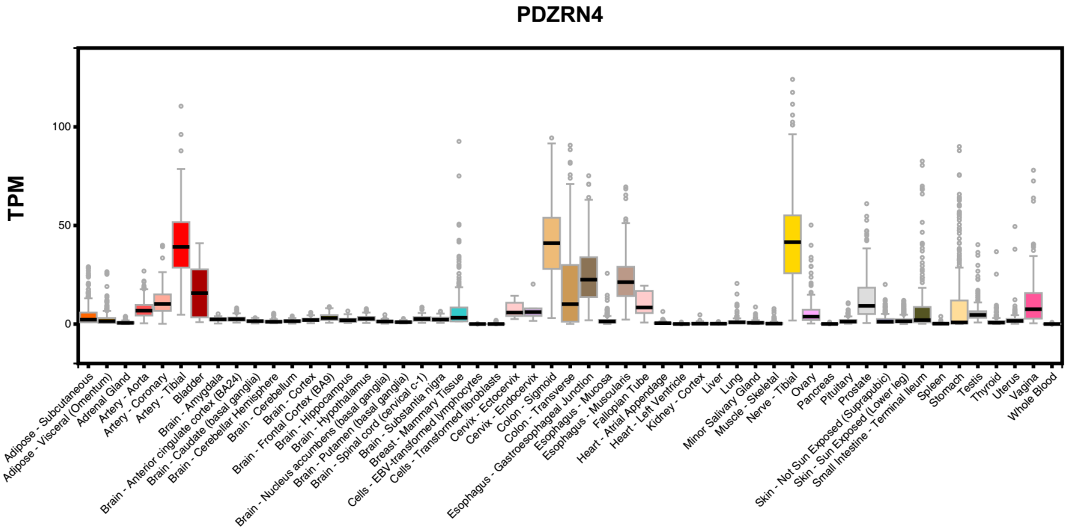

Supplementary Figure 12. Mutational lolliplot of *PDZRN4* (A) and *SNC2A* (B). For each gene, the mutations of our study (up) and COSMIC (down) are presented as a circle with different colors according to their function annotation. The protein domains are presented according to Pfam databases (https://pfam.xfam.org/).

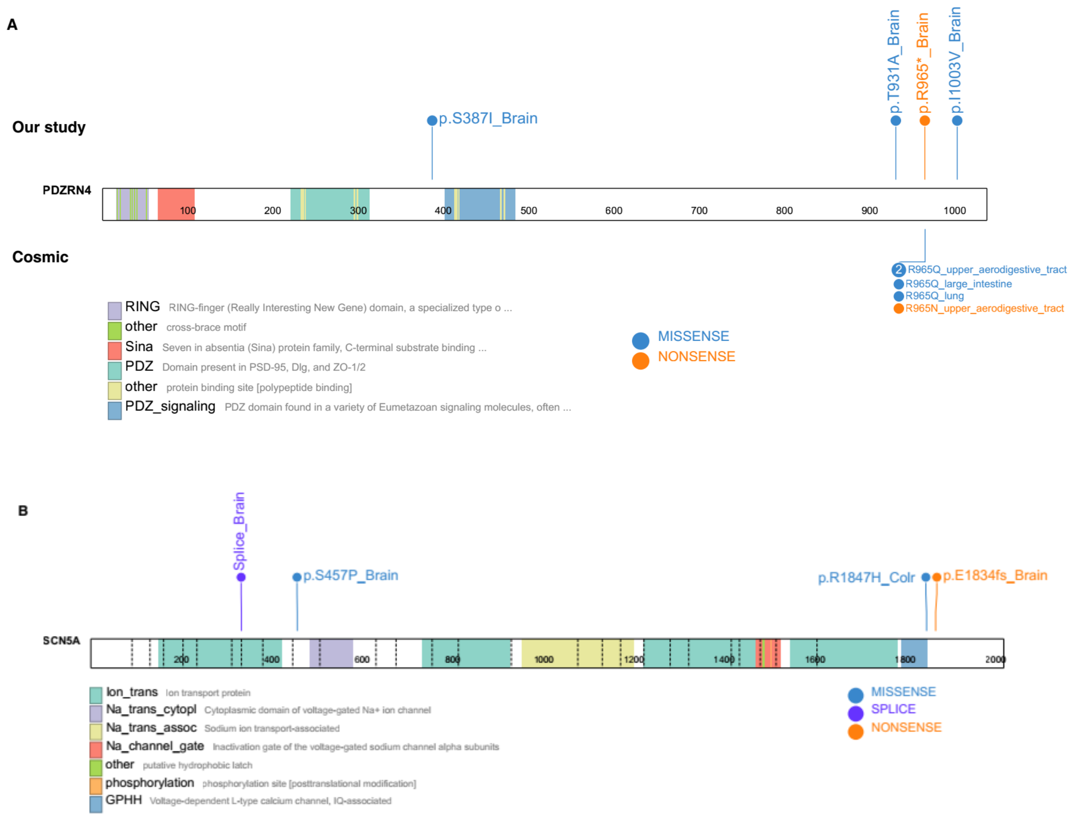

Supplementary Figure 13. Validation of brain metastases specific recurrent mutations in *SCN7A*. The results of sanger sequencing of brain metastasis (A), primary and normal samples (B) were presented.

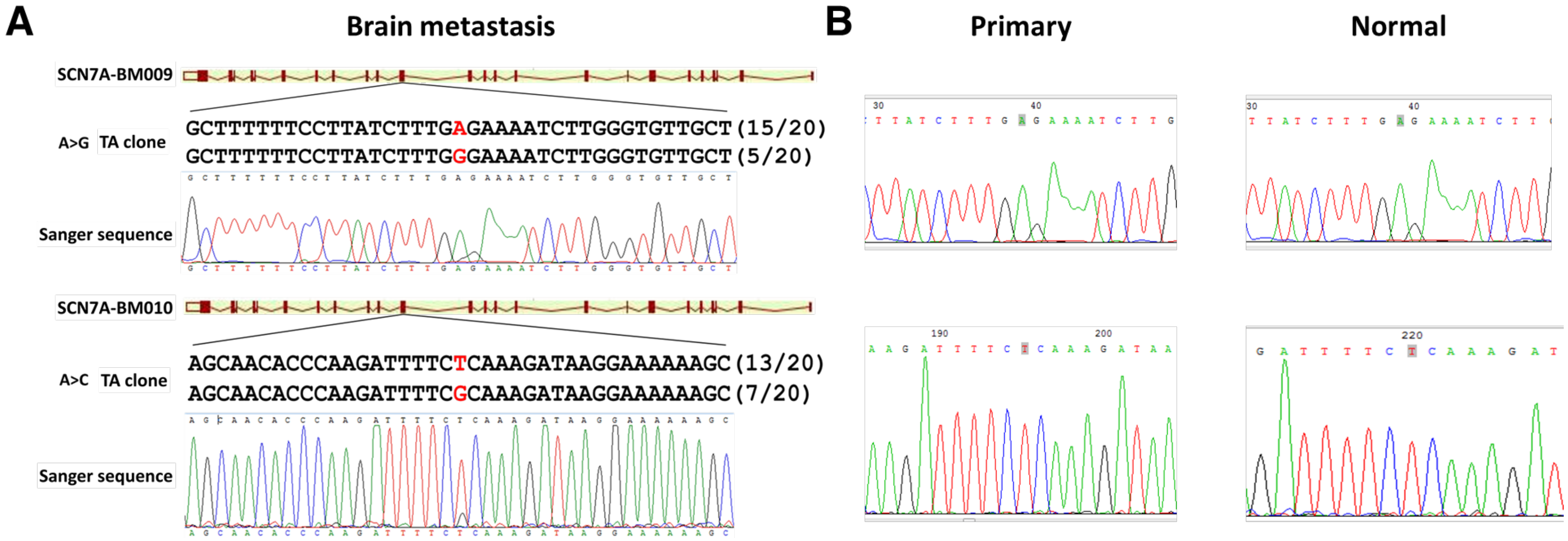

Supplementary Table 1. Summary of clinical information of 19 CRC patients with brain metastases and 5 CRC patients with liver metastases.

ID	Gender	Primary Site	Age of Diagnosis of Primary Tumor	Brain Met Progression Free Survival in Years (Time between diagnosis of primary tumor and sequenced brain metastasis)	Age of Death	Metastasis sites before brain met (0=no；1=liver; 2=lung; 3:other)	Radiotherapy prior to brain met (1=yes; 0=no)	Chemotherapy prior to brain metastasis (1=yes; 0=no)	Targeted therapy prior to brain metastasis (1=yes; 0=no)	Immunotherapy prior to brain metastasis (1=yes; 0=no)	Brain Radiation Prior to Metastasis Resection (1=yes; 0=no)	Drinking history (1=yes; 0=no)	Smoking history (1=yes; 0=no)	Other brain metastases(1=yes; 0=no)	Stage of diagnosis of primary tumor (T N Mstage)	Nodal involvement at diagnosis of primary tumor	Grade of primary	Vascular invasion(1=yes; 0=no)	perineural invasion (1=yes; 0=no)	Size of primary tumor at diagnosis (cm)	chemotherapy prior to brain surgery(1=Oxaliplatin; 2= Irinotecan; 3 = Fluorouracil;4= Bevacizumab; 5= Cetuximab; 6=Oral targeted drugs)	AJCC Stage of primary tumor	Platform
BM001	F	rectum	65.1	3.75	69.2	0	0	1	0	0	0	0	0	0	T3N0M0	0/15	Well differentiated	0	0	1.5 × 1.4 × 0.8	1,2,3	IIA	WES
BM002	F	rectum	61.5	3.33	66.4	2	1	1	0	0	0	0	1	1	T3N2aM0	4/6	Moderately to poorly differentiated	1	1	3.2 × 3.5 × 0.6	1,2,3	IIIB	WES
BM010	F	colon（right)	59	2.9	63	0	0	1	0	0	0	0	0	0	T3N0M0	0/18	Moderately differentiated	0	1	3.5 × 2.5 × 0.5	1,2,3	IIA	WES
BM008	M	rectum	63	6.1	69.9	2	1	1	0	0	0	0	1	1	T3N1bM0	3/3	Moderately differentiated	0	0	3 .2 × 2.2 × 1.7	1,2,3	IIIB	WES
BM006	F	rectum	58.7	3.1	63.5	1.2	1	1	0	0	0	0	0	1	T3N0M0	0/3	Moderately differentiated	0	0	6.6 × 4.8 × 3.7	1,2,3	IIA	WES
BM009	M	colon(right)	75.8	5.2	81.5	2	0	1	0	0	0	0	1	0	T3N0M0	0/38	Moderately differentiated	0	0	9 × 9.2 × 7.1	1,2,3	IIA	WES
BM005	M	rectum	55.6	2.8	59.4	2	1	1	0	0	0	1	1	0	T3N2aM0	5/15	Moderately to poorly differentiated	1	0	5 × 4.2 × 2.1	1,2,3	IIIB	WES
BM007	F	rectum	40.1	3.8	45.1	2	1	1	0	0	0	0	0		T3N1aM0	1/4	Moderately to well differentiated	0	0	3.3 × 2.2 × 2.1	1,2,3	IIIB	WES
BM003	M	rectum	38.3	0.5	40.1	3	1	1	0	0	0	0	0	1	T4aN2bM0	24/24	Poorly differentiated	1	1	3.2 × 2 × 1.7	1,2,3	IIIC	WES
BM011	F	rectum	53.2	2.1	56.3	3	0	1	1	0	0	0	0	0	T4aN0M0	0/8	Moderately to poorly differentiated	0	1	2.5 × 2 × 1.5	1,2,3,5	IIB	WES
BM004	M	colon(right)	63.5	2.5	67.2	1.2	0	1	0	0	0	0	1	0	T4aN1cM0	0/14	Moderately to poorly differentiated	0	0	10 × 6 × 2	1,2,3	IIIB	WES
BM012	F	colon(left)	56.5	1.5	58.7	2.3	0	1	0	0	0	0	0	0	T3N1cM0	0/8	Moderately differentiated	0	1	2.5 × 2 × 2	1,2,3	IIIB	WGS
BM014	M	rectum	79.2	1.9	Alive	2	0	1	1	0	0	0	0	0	T3N1M0	1/8	Moderately to poorly differentiated	1	1	3 × 2 × 0.6	1,2,3,4	IIIB	WGS
BM017	M	colon(right)	54.2	1.2	57.9	2	0	1	1	0	0	1	0	0	T2N0M0	0/19	Moderately differentiated	0	0	1.5 × 1 × 1	1,2,3,4	IA	WGS
BM019	M	rectum	56.3	2.8	60.8	2	1	1	0	0	0	1	1	0	T2N0M0	0/12	Moderately differentiated	0	0	2.6 × 2.5 × 1	1,2,3	IA	WGS
BM016	F	rectum	54.7	0.6	55.5	2	0	1	0	0	0	0	0	0	T3N2bM0	7/8	Moderately differentiated	1	1	5 × 3.4 × 3.1	1,2,3	IIIC	WGS
BM018	M	rectum	47.2	6.8	54.9	2	1	1	1	0	0	1	1	0	T3N1bM0	2/8	Moderately differentiated	0	0	5 × 4.5 × 1.5	1,2,3,4	IIIB	WGS
BM015	M	rectum	55.1	1.2	Alive	2.3	0	1	1	0	0	1	1	0	T4aN2bM0	2/8	Moderately to poorly differentiated	1	0	6 × 4 × 1	1,2,3,4,6	IIIC	WGS
BM013	M	rectum	51.2	4.1	56.7	2	1	1	0	0	0	1	0	0	T3N1bM0	1/8	Moderately differentiated	1	0	5.2 × 3.5 × 1.2	1,2,3	IIIB	WGS
BM020	F	colon(right)	78.6	2.3	81.5	3	0	1	1	0	0	0	0	0	T3N2aM0	5/25	Moderately to poorly differentiated	1	1	6×5.5×1.4	1,2,3,4	IIIb	RNA-Seq
BM021	M	rectum	60.1	1.5	Alive	2	1	1	0	0	0	1	1	0	T3N1MO	1/25	Moderately differentiated	0	0	6×3.4×1.1	1,2,3	IIIb	RNA-Seq
LM001	M	colon(left)	63.2	/	Alive	/	0	1	1	0	/	1	1	/	T3N2aM1a	2/8	Moderately to poorly differentiated	1	0	4×3×2	1,2,3	IV	WES
LM002	F	rectum	65.1	/	Alive	/	1	1	1	0	/	0	0	/	T3N2bM1a	3/8	Moderately to poorly differentiated	0	1	5.5×4.5×0.8	1,2,3	IV	WES
LM003	M	colon(left)	58.5	/	Alive	/	0	1	0	0	/	1	1	/	T4aN2aM1a	2/8	Moderately differentiated	0	1	2.5×2.2×0.8	1,2,3	IV	WES
LM004	F	colon(left)	75.2	/	Alive	/	0	1	0	0	/	0	0	/	T4aN1bM1a	1/8	Moderately to poorly differentiated	0	1	4×3×2.5	1,2,3	IV	WES
LM005	M	colon(left)	69.8	/	Alive	/	0	1	0	0	/	1	1	/	T3N0M1a	0/13	Moderately differentiated	0	1	4×2.5×0.5	1,2,3	IV	WES

Supplementary Table 2. Mutation number and purity in all Brain metastasis patients.

ID	Group	purity	Mutation Number	Mutation rate (/MB)
BM001	Brain	0.88	349	2.97
BM001	Primary	1	446	3.45
BM001	Primary	0.69	269	2.74
BM008	Brain	1	376	3.48
BM008	Primary	1	473	4.44
BM008	Primary	1	95	0.78
BM009	Brain	0.5	472	4.32
BM009	Primary	0.81	343	2.99
BM009	Primary	0.8	434	4.79
BM003	Brain	0.89	259	2.34
BM003	Primary	0.45	235	2.01
BM003	Primary	0.46	159	1.30
BM011	Brain	0.81	261	2.29
BM011	Primary	0.43	86	0.68
BM010	Brain	0.56	473	3.97
BM010	Primary	0.82	384	3.33
BM010	Primary	0.75	398	3.56
BM007	Brain	0.84	371	3.23
BM007	Primary	0.53	225	2.00
BM006	Brain	0.79	345	2.98
BM006	Primary	0.57	267	2.31
BM006	Primary	0.66	245	2.14
BM004	Brain	0.97	295	2.42
BM004	Primary	0.46	197	1.92
BM004	Primary	0.9	262	2.43
BM002	Brain	0.69	308	2.56
BM002	Primary	0.33	362	3.06
BM002	Primary	0.37	268	2.36
BM005	Brain	0.73	193	1.69
BM005	Primary	0.79	253	2.21
BM005	Primary	0.6	212	1.84
BM012	Brain	0.85	9460	3.34
BM012	Primary	0.73	13083	4.65
BM013	Brain	0.92	21900	7.79
BM013	Primary	0.23	28238	11.37
BM014	Brain	0.74	13934	4.91
BM014	Primary	0.5	10549	3.71
BM015	Brain	0.84	33312	11.81
BM015	Primary	0.5	18276	6.45
BM016	Brain	0.75	9330	3.30
BM016	Primary	0.14	7457	2.63

BM017	Brain	0.89	20072	7.09
BM017	Primary	0.11	11389	4.01
BM018	Brain	0.31	33843	11.95
BM018	Primary	0.17	12445	4.47
BM019	Brain	0.94	15526	5.95
BM019	Primary	0.15	18803	6.69

Supplementary Table 3. The mean proportion of 30 COSMIC signatures in metastasis CRC (WES), metastasis CRC (WGS) and TCGA patients

Cosmic Signatures	Metastasis CRC (WES)		Metastasis CRC (WGS)		TCGA CRC	
	Brain	Primary	Brain	Primary	MSI	MSS
Signature 1	0.42	0.76	0.26	0.35	0.33	0.47
Signature 3	0.13	0.04	0.07	0.02	0.00	0.01
Signature 17	0.12	0.02	0.21	0.03	0.00	0.01
Signature 6	0.10	0.03	0.02	0.03	0.44	0.05
Signature 18	0.04	0.02	0.06	0.07	0.00	0.02
Signature 15	0.03	0.02	0.02	0.01	0.09	0.03
Signature 4	0.02	0.01	0.00	0.00	0.00	0.01
Signature 13	0.02	0.01	0.00	0.00	0.00	0.00
Signature 24	0.02	0.00	0.00	0.00	0.00	0.01
Signature 25	0.01	0.00	0.00	0.02	0.00	0.00
Signature 28	0.01	0.01	0.01	0.00	0.00	0.00
Signature 2	0.01	0.01	0.01	0.03	0.00	0.00
Signature 7	0.01	0.02	0.00	0.01	0.00	0.01
Signature 8	0.01	0.00	0.13	0.17	0.00	0.00
Signature 29	0.01	0.00	0.00	0.00	0.00	0.00
Signature 10	0.01	0.01	0.01	0.01	0.00	0.03
Signature 11	0.01	0.01	0.00	0.01	0.00	0.00
Signature 19	0.01	0.01	0.00	0.00	0.00	0.00
Signature 22	0.01	0.00	0.00	0.00	0.00	0.00
Signature 21	0.00	0.00	0.00	0.00	0.01	0.01
Signature 20	0.00	0.00	0.00	0.00	0.01	0.03
Signature 23	0.00	0.00	0.00	0.00	0.00	0.00
Signature 9	0.00	0.00	0.14	0.09	0.00	0.00
Signature 27	0.00	0.01	0.01	0.05	0.00	0.00
Signature 5	0.00	0.00	0.02	0.06	0.00	0.00
Signature 12	0.00	0.00	0.01	0.03	0.04	0.27
Signature 14	0.00	0.00	0.00	0.00	0.00	0.00
Signature 16	0.00	0.00	0.00	0.01	0.00	0.00
Signature 26	0.00	0.00	0.00	0.00	0.05	0.00
Signature 30	0.00	0.00	0.00	0.00	0.00	0.00
