# Supplementary material for: Genomic signatures reveal DNA damage response deficiency in colorectal cancer brain metastases
Source: Nat Commun. 2019 Jul 18;10:3190. doi: 10.1038/s41467-019-10987-3 (PMC6639368; doi:10.1038/s41467-019-10987-3)
Supplement: Supplementary file 3 — Description of Additional Supplementary Files [file 41467_2019_10987_MOESM3_ESM.pdf]

Description of Additional Supplementary Files

File Name: Supplementary Data 1
Description: The mutation patterns of different 30 COSMIC mutational signatures.

File Name: Supplementary Data 2
Description: Differential expression analysis of mismatch repair and homologous recombination genes between brain metastasis and primary CRC tissues.

File Name: Supplementary Data 3
Description: All mutations in DDR genes.
