# Supplementary material for: Genomic signatures reveal DNA damage response deficiency in colorectal cancer brain metastases
Source: Nat Commun. 2019 Jul 18;10:3190. doi: 10.1038/s41467-019-10987-3 (PMC6639368; doi:10.1038/s41467-019-10987-3)
Supplement: Supplementary file 7 — Reporting Summary [file 41467_2019_10987_MOESM7_ESM.pdf]

nature research

Corresponding author(s): Yanhong Gu

Last updated by author(s): May 24, 2019

Reporting Summary

Nature Research wishes to improve the reproducibility of the work that we publish. This form provides structure for consistency and transparency in reporting. For further information on Nature Research policies, see Authors & Referees and the Editorial Policy Checklist.

Statistics

For all statistical analyses, confirm that the following items are present in the figure legend, table legend, main text, or Methods section.

n/a	Confirmed	
☐	☒	The exact sample size (*n*) for each experimental group/condition, given as a discrete number and unit of measurement
☐	☒	A statement on whether measurements were taken from distinct samples or whether the same sample was measured repeatedly
☐	☒	The statistical test(s) used AND whether they are one- or two-sided<br>*Only common tests should be described solely by name; describe more complex techniques in the Methods section.*
☐	☒	A description of all covariates tested
☐	☒	A description of any assumptions or corrections, such as tests of normality and adjustment for multiple comparisons
☐	☒	A full description of the statistical parameters including central tendency (e.g. means) or other basic estimates (e.g. regression coefficient) AND variation (e.g. standard deviation) or associated estimates of uncertainty (e.g. confidence intervals)
☐	☒	For null hypothesis testing, the test statistic (e.g. *F*, *t*, *r*) with confidence intervals, effect sizes, degrees of freedom and *P* value noted<br>*Give P values as exact values whenever suitable.*
☒	☐	For Bayesian analysis, information on the choice of priors and Markov chain Monte Carlo settings
☒	☐	For hierarchical and complex designs, identification of the appropriate level for tests and full reporting of outcomes
☒	☐	Estimates of effect sizes (e.g. Cohen's *d*, Pearson's *r*), indicating how they were calculated

Our web collection on statistics for biologists contains articles on many of the points above.

Software and code

Policy information about availability of computer code

Data collection	The whole-exome sequencing data were collected using the Illumina HiSeq 2500 sequencing platform. The whole-genome sequencing data were collected using the Illumina HiSeq X Ten instrument run in the standard mode in WuXi NextCODE (Shanghai) Co., Ltd. RNA purification, reverse transcription, library construction and sequencing were performed at WuXi NextCODE at Shanghai according to the manufacturer's instructions (Illumina).
Data analysis	Read sequences were mapped to the human reference genome (GRCh37) using Burrows-Wheeler Aligner (BWA-MEM v0.7.15-r1140) with the default parameters, and duplicates were marked and discarded using Picard (v1.70) (http://broadinstitute.github.io/picard). After alignment by BWA, the reads were subjected to local realignment and recalibration using the Genome Analysis Toolkit (GATK v3.5). Somatic substitutions and indels were detected using the MuTect2 mode in GATK on the GRCh37 genome build following the best practices for somatic SNV/indel calling (https://software.broadinstitute.org/gatk/best-practices/). Briefly, the algorithms compared the tumor with the matched normal sample to exclude germline variants. RNA reads were generated, aligned to the GENCODE v19 genome assembly with STAR v2.4.1 and quantified using featureCounts v1.5.0. The raw read counts were normalized with DESeq2 followed by differential expression analysis.

For manuscripts utilizing custom algorithms or software that are central to the research but not yet described in published literature, software must be made available to editors/reviewers. We strongly encourage code deposition in a community repository (e.g. GitHub). See the Nature Research guidelines for submitting code & software for further information.

Data

Policy information about availability of data

All manuscripts must include a data availability statement. This statement should provide the following information, where applicable:

- Accession codes, unique identifiers, or web links for publicly available datasets
- A list of figures that have associated raw data
- A description of any restrictions on data availability

The whole-exome sequencing data and the whole-genome sequencing data have been deposited in the European Genome-phenome Archive (EGA) at the EMBL-

European Bioinformatics Institute under accession number EGAD00001005030 (https://ega-archive.org/datasets/EGAD00001005030). The RNA-Seq data have been deposited at figshare (https://doi.org/10.6084/m9.figshare.8180384.v1). All the other data supporting the findings of this study are available within the article and its supplementary information files and from the corresponding author upon reasonable request.

Field-specific reporting

Please select the one below that is the best fit for your research. If you are not sure, read the appropriate sections before making your selection.

- [x] Life sciences
- [ ] Behavioural & social sciences
- [ ] Ecological, evolutionary & environmental sciences

For a reference copy of the document with all sections, see nature.com/documents/nr-reporting-summary-flat.pdf

Life sciences study design

All studies must disclose on these points even when the disclosure is negative.

Sample size	We conducted WES on 42 tissues from 11 CRC patients with brain metastases and WGS on 24 tissues from 8 CRC patients with brain metastases.
Data exclusions	The patients received prior treatment before colorectal cancer surgery were excluded.
Replication	Not applicable
Randomization	Not applicable
Blinding	Not applicable

Reporting for specific materials, systems and methods

We require information from authors about some types of materials, experimental systems and methods used in many studies. Here, indicate whether each material, system or method listed is relevant to your study. If you are not sure if a list item applies to your research, read the appropriate section before selecting a response.

Materials & experimental systems

n/a	Involved in the study	
☒	☐	Antibodies
☒	☐	Eukaryotic cell lines
☒	☐	Palaeontology
☒	☐	Animals and other organisms
☐	☒	Human research participants
☒	☐	Clinical data

Methods

n/a	Involved in the study	
☒	☐	ChIP-seq
☒	☐	Flow cytometry
☒	☐	MRI-based neuroimaging

Human research participants

Policy information about studies involving human research participants

Population characteristics	The clinical characteristics of the 19 patients are summarized in Supplementary Table 1.
Recruitment	All participants were recruited from hospital and all participants provided written informed consent for genetic analysis.
Ethics oversight	The study protocol was reviewed and approved by the Ethics Committee of Jiangsu Province Hospital, the First Affiliated Hospital of Nanjing Medical University.

Note that full information on the approval of the study protocol must also be provided in the manuscript.
